# Age-related changes in sleep spindles characteristics during daytime recovery following a 25-hour sleep deprivation

**DOI:** 10.3389/fnhum.2015.00323

**Published:** 2015-06-03

**Authors:** T. Rosinvil, M. Lafortune, Z. Sekerovic, M. Bouchard, J. Dubé, A. Latulipe-Loiselle, N. Martin, J. M. Lina, J. Carrier

**Affiliations:** ^1^Center for Advanced Research in Sleep Medicine, Hôpital du Sacré-Coeur de MontréalMontréal, QC, Canada; ^2^Department of Psychology, Université de MontréalMontréal, QC, Canada; ^3^Research Center, Institut Universitaire Gériatrique de MontréalMontréal, QC, Canada; ^4^Department of Electrical Engineering, École de Technologie SupérieureMontréal, QC, Canada

**Keywords:** aging, sleep spindles, circadian process, sleep loss, homeostatic sleep pressure

## Abstract

**Objectives**: The mechanisms underlying sleep spindles (~11–15 Hz; >0.5 s) help to protect sleep. With age, it becomes increasingly difficult to maintain sleep at a challenging time (e.g., daytime), even after sleep loss. This study compared spindle characteristics during daytime recovery and nocturnal sleep in young and middle-aged adults. In addition, we explored whether spindles characteristics in baseline nocturnal sleep were associated with the ability to maintain sleep during daytime recovery periods in both age groups.

**Methods**: Twenty-nine young (15 women and 14 men; 27.3 y ± 5.0) and 31 middle-aged (19 women and 13 men; 51.6 y ± 5.1) healthy subjects participated in a baseline nocturnal sleep and a daytime recovery sleep after 25 hours of sleep deprivation. Spindles were detected on artifact-free Non-rapid eye movement (NREM) sleep epochs. Spindle density (nb/min), amplitude (μV), frequency (Hz), and duration (s) were analyzed on parasagittal (linked-ears) derivations.

**Results**: In young subjects, spindle frequency increased during daytime recovery sleep as compared to baseline nocturnal sleep in all derivations, whereas middle-aged subjects showed spindle frequency enhancement only in the prefrontal derivation. No other significant interaction between age group and sleep condition was observed. Spindle density for all derivations and centro-occipital spindle amplitude decreased whereas prefrontal spindle amplitude increased from baseline to daytime recovery sleep in both age groups. Finally, no significant correlation was found between spindle characteristics during baseline nocturnal sleep and the marked reduction in sleep efficiency during daytime recovery sleep in both young and middle-aged subjects.

**Conclusion**: These results suggest that the interaction between homeostatic and circadian pressure modulates spindle frequency differently in aging. Spindle characteristics do not seem to be linked with the ability to maintain daytime recovery sleep.

Non-rapid-eye movement (NREM) sleep is a global brain process commonly defined by an absence of interaction with the environment, altered awareness, reduced external information processing and enhanced cortical synchronization. High levels of cortical synchronization during slow-wave sleep (SWS or N3 NREM sleep) is characterized by high-amplitude (>75 mV) electroencephalographic (EEG) slow waves (<4 Hz; SW). SW have two phases at the cellular level: a hyperpolarization phase (surface EEG SW negative phase), during which cortical neurons are mostly silent, and a depolarization phase (surface EEG SW positive phase), during which most cortical neurons fire intensively (Steriade, 2006). Sleep spindles (waxing and waning EEG waves of 12–15 Hz; >0.5 s) occur mostly during N2 NREM sleep but still persist in N3 NREM sleep to be eventually replaced by SWs. Hence, several observations support a reciprocal relationship between sleep spindles and SW in NREM sleep (Dijk et al., 1993; Steriade et al., 1993; for a review: De Gennaro and Ferrara, 2003).

Aging is associated with less time asleep, more frequent awakenings of longer duration and shallower sleep (Buysse et al., 1992; Hoch et al., 1994; Landolt et al., 1996; Carrier et al., 1997, 2001; Landolt and Borbély, 2001). These changes are part of the normal aging process and occur gradually during the middle years of life (Carrier et al., 2001). Moreover, NREM sleep changes drastically in the middle years of life through a substantial reduction in SWS and an increase in lighter NREM sleep stages (Hoch et al., 1994; Landolt et al., 1996; Carrier et al., 1997). Studies have shown considerable changes in NREM sleep from age 20 to 60 years, including significant decreases in slow-wave activity (SWA; i.e., spectral power between 0.5–4.5 Hz) and low sigma activity (spectral power between 13–14 Hz), during NREM (Carrier et al., 2001; Landolt and Borbély, 2001). Our group has also shown that middle-aged subjects exhibit lower density and amplitude of SW and spindles when compared to younger participants, especially in prefrontal/frontal brain areas (Carrier et al., 2011; Lafortune et al., 2012; Martin et al., 2013).

The sleep-wake cycle is regulated by the interaction between the homeostatic and the circadian processes (Dijk and Czeisler, 1994). The homeostatic process represents the sleep pressure accumulated by the time spent awake and dissipated during a sleep episode (Achermann et al., 1993). In humans, the intensity and dynamics of slow wave activity (SWA; spectral power between 0.5–4 Hz in NREM) model the time course of the homeostatic process (i.e., more time awake produces more SWA, whereas more time asleep is associated with less SWA; Achermann et al., 1993). A few studies showed lower rebound of SWA as well as SW density and amplitude after sleep deprivation in middle-aged and older subjects when compared to younger participants, particularly in anterior brain areas (Gaudreau et al., 2001a; Münch et al., 2004; Carrier et al., 2009; Lafortune et al., 2012). The latter results suggest that there is a reduction in homeostatic sleep pressure as age increases starting in the middle years of life. On the other hand, a biological “clock” located in the suprachiasmatic nucleus controls the circadian process of sleep regulation. Circadian wake propensity increases during the day and maximizes in the evening (Czeisler et al., 1980; Zulley et al., 1981; Lavie, 1985). Studies have shown that sleep in middle-aged and older subjects is particularly vulnerable to circadian phases of high wake propensity, which means that it is more difficult with aging to maintain sleep at the “wrong” circadian phase (e.g., in the daytime), even after sleep deprivation (Cajochen et al., 1999; Gaudreau et al., 2001b). The mechanisms underlying this stronger enhancement of wakefulness during daytime recovery sleep in middle-aged and older participants compared to younger subjects remain unknown. Recently, we tested whether age-related modifications in SW could be linked to enhanced wakefulness during daytime recovery sleep, but none of the SW characteristics at baseline were associated with daytime recovery sleep efficiency in young and middle-aged subjects (Lafortune et al., 2012).

One of the functional roles attributed to sleep spindles is to prevent afferent signals from being transmitted to the cortex, thus allowing cortical unresponsiveness to stimulation during sleep (Steriade et al., 1993; Steriade, 1994, 2006; Bazhenov et al., 1999; Born et al., 2002; Czisch et al., 2002; Dang-Vu et al., 2011). Hence, age-related changes in spindles may be linked to the ability to maintain sleep at an abormal circadian phase. Interestingly, spindle characteristics are also regulated by the interaction between the homeostatic and the circadian processes. Compared to conditions of lower homeostatic sleep pressure prior to nocturnal sleep, studies have shown a reduction in spindle density and in spindle mean frequency under higher sleep homeostatic pressure in young subjects (Curcio et al., 2003; Knoblauch et al., 2003a). However, to our knowlegde, no study has evaluated age-related effects of sleep deprivation on spindles. Studies have also reported lower spindle density and higher spindle mean frequency when sleep occurred at a circadian time corresponding to daytime in comparison to night-time (Wei et al., 1999; Knoblauch et al., 2003b, 2005). Importantly, this circadian modulation of spindles is reduced in older subjects when compared to younger subjects (Wei et al., 1999; Knoblauch et al., 2005).

The main aim of this study is to compare sleep spindles characteristics between baseline nocturnal sleep and daytime recovery sleep after 25 h of total sleep deprivation in both young and middle-aged subjects. We also evaluated whether sleep spindles are associated with the ability to maintain sleep during daytime recovery sleep. We predict that middle-aged subjects will have a lower reduction of spindles during daytime recovery sleep compared to younger subjects and that higher spindle density during baseline sleep will be associated with a smaller decrease in sleep efficiency during daytime recovery sleep in young and older subjects.

## Methods

### Subjects and Procedure

Twenty-nine young (15 women and 14 men; 20–38 years old, mean = 27.3 years, SD = 5.0) and 31 middle-aged (19 women and 13 men, 40–60 years old, mean = 51.6 years, SD = 5.1) healthy subjects were recruited for this study. Data from participants were drawn from two studies conducted between 1999 and 2006 in our laboratory, all following similar recording procedures and free from active pharmacological manipulation (Gaudreau et al., 2001b; Carrier et al., 2009). All subjects signed an informed consent form and received monetary compensation for their participation. All research studies were approved by the ethical committee of the Hôpital du Sacré-Coeur de Montréal.

A semi-structured interview using a homemade questionnaire was performed to exclude potential subjects who smoked, used sleep-affecting medication and reported sleep complaints or unusual sleep duration (i.e., <7 h and >9 h). Participants who engaged in night work or transmeridian travel 3 months prior to the study were also excluded. No subjects reported neurological or psychiatric illness history using our homemade questionnaire, nor showed indication of depression (Beck Depression Inventory, short version >3 or long version >9; Beck and Steer, 1987). Moreover, to rule out any significant medical condition, certified physicians evaluated blood sample analysis (complete blood count, serum chemistry, including hepatic and renal functions; prolactin level; testosterone level in men; and estrogen, follicle stimulating hormone (FSH) and luteinizing hormone levels in women) and urinalysis results. Perimenopausal women and women using hormonal contraception or receiving hormonal replacement therapy were excluded. Premenopausal women reported regular menstrual cycles (25–32 days) in the year preceding the experiment, had no vasomotor complaints (i.e., night sweats, hot flashes) and showed low FSH levels (<20 iU/L). All postmenopausal women reported an absence of menses in the past year and showed high FSH levels (>20 iU/L).

Prior to data acquisition, all subjects underwent a polysomnographic (PSG) adaptation and screening night; including nasal/oral thermistor and an electromyogram (EMG) leg electrode recordings to screen for sleep disturbances. The presence of sleep disorders such as sleep apneas, hypopneas and periodic leg movements (index per hour >10) resulted in the participant’s exclusion.

All subjects came to the laboratory for a baseline nocturnal sleep episode (BSL). The following night, subjects were sleep deprived. A morning recovery sleep episode (REC) was initiated one hour after their habitual wake time (after 25 h of wakefulness). During the night of sleep deprivation, all subjects remained awake in a semi-recumbent position in dim light (<15 lux) until the next morning. Bedtime and wake time in the laboratory were determined using averaged regular schedules obtained from sleep diary entries (recorded 7 days prior to BSL).

### Polysomnographic Recordings

PSG recordings included EEG electrodes (10–20 system, referential montage with linked ears), chin EMG and left and right electrooculography (EOG). PSG was recorded using a Grass Model 15 amplifier system (gain 10,000; bandpass 0.3–100 HZ). Signals were digitalized at a sampling rate of 256 Hz using commercial software (Harmonie, Stellate System). Sleep stages were visually scored on C3 in 20-s epochs on a computer screen according to standard criteria (Rechtschaffen and Kales, 1968). EEG artifacts were detected automatically (Brunner et al., 1996) and then inspected visually to ensure appropriate rejection from analysis.

### Automatic Algorithm Detection of Sleep Spindles

Sleep spindles were detected automatically on artifact-free NREM epochs for left and right parasagittal scalp derivations (i.e., Fp1, F3, C3, P3, O1 and Fp2, F4, C4, P4, O2). EEG data were first bandpass filtered from 11 to 15 Hz with a linear phase Finite Impulse Response filter (−3 dB at 11.1 and 14.9 Hz). Forward and reverse filtering was performed to obtain zero-phase distortion and to double the filter order. The root mean square (RMS) of the filtered signal was then calculated with a 0.25 s time window and thresholded at its 95th percentile (Schabus et al., 2007). A spindle was identified when at least two consecutive RMS time-points exceeded the threshold, reaching duration criterion (0.5 s; no superior limit but 98% of spindles were ≤1 s). Four spindle characteristics were derived: density (number of spindles/minutes of NREM sleep, expressed in nb/min), amplitude (peak-to-peak difference in voltage, expressed in μV), frequency (number of cycles/second, expressed in Hz), and duration (expressed in seconds). Spindle characteristics were assessed over the entire night. Spindle characteristics from left and right electrodes were averaged together (prefrontal: FP1–FP2, Frontal: F3–F4, Central: C3–C4, Parietal: P3–P4, Occipital: O1–O2).

### Statistical Analyses

#### Preliminary Analyses

To evaluate possible interaction between sex, age and sleep conditions, 3-way mixed design analysis of variance (ANOVA) with two independent factors (age groups: young and middle-aged; sex groups: men and women) and one repeated measure (2 sleep conditions: BSL, REC) were performed on PSG variables and spindle characteristics for each topographical site (prefrontal, frontal, central, parietal, occipital). No significant interactions between age group, sex and sleep condition were found for PSG characteristics and all spindle characteristics, except for sleep spindle density in the central area (*F*_(1,57)_ = 5.64, *p* = 0.02). *Post hoc* analysis revealed an age group by sleep condition interaction for women (*F*_(1,32)_ = 5.14, *p* = 0.03) and not for men (*F*_(1,25)_ = 1.39, *p* = 0.25). Middle-aged women showed a stronger decrease in spindle density in the central area compared to young women. In men, spindle density was lower from BSL to REC in both age group resulting in only a main effect of sleep condition (*F*_(1,25)_ = 72.58, *p* < 0.0001). Consequently, data from men and women were pooled together, except for spindle density, which was analyzed separately in men and women.

#### Analyses

Two-way ANOVAs with one independent factor (2 age groups) and one repeated measure (2 sleep conditions: BSL, REC) were performed on PSG sleep variables. Mixed ANOVAs with one independent factor (2 age groups) and two repeated measures (2 sleep conditions: BSL, REC; 5 derivations: Prefrontal, Frontal, Central, Parietal and Occipital) were performed for each spindle characteristic.

*P* values for repeated measures with more than two levels were adjusted for sphericity with Huynh-Feldt corrections, but original degrees of freedom were reported. Differences in main effects and in interactions were assessed with *post hoc* multiple mean comparisons, and effect size (ES) were measured using the partial ETA square and Wilk’s Lambda partial ETA square when applicable. Results were considered significant when *p* ≤ 0.05.

Pearson correlations were performed between all-night spindle characteristics during baseline sleep and the change in sleep efficiency between BSL and REC sleep (absolute and % in change) in the young and the middle-aged groups separately and in the two groups pooled together with age as a control variable. In these analyses, we applied a more severe level of significance (i.e., *p* ≤ 0.01) to correct for multiple comparisons.

## Results

### Sleep Architecture

Sleep efficiency and duration was lower during REC sleep as compared to BSL sleep in both age groups. However, this reduction of sleep efficiency and duration was more prominent in the middle-aged than in the young subjects. SWS was higher during REC sleep compared to BSL sleep, but this effect was less prominent in middle-aged compared to young subjects. As for sleep latency, % of stages 2 and REM sleep, they were all lower in REC sleep when compared to BSL sleep. Finally, middle-aged subjects showed a higher percentage of stage 2 sleep in comparison to younger participants (see Table 1 for all effects).

**Table 1:** **Polysomnographic variables for young and middle-aged subjects in both sleep conditions**.

	Young		Middle-Aged		Age effect	Condition effect	Age × Condition interaction
PSG variables	BSL	REC	BSL	REC	*F(p)*	*F(p)*	*F(p)*
**Sleep latency (min)**	9.2 (5.8)	3.5 (3.6)	10 (5.9)	5.2 (4.8)	n.s.	*F* = 46.5 (*p* < 0.0001)	n.s.
**Sleep duration (min)**	437.3 (38.5)	377.9 (65.1)	428.8 (46.2)	335.1 (59.7)	*F* = 5.5 (*p* = 0.02)	*F* = 83.5 (*p* < 0.0001)	*F* = 4.3 (*p* = 0.04)
**Efficiency (%)**	91.1 (5.7)	79.8 (14.4)	87.7 (6.1)	69.2 (13)	*F* = 10.2 (*p* = 0.002)	*F* = 89.2 (*p* < 0.0001)	*F* = 5.1 (*p* = 0.02)
**Stage 1 (%)**	7.7 (3.7)	7.9 (5.4)	7.9 (3.4)	8.6 (3.6)	n.s.	n.s.	n.s.
**Stage 2 (%)**	59.8 (5.6)	56.5 (9.5)	66.4 (5.6)	65.8 (6.7)	*F* = 25.1 (*p* < 0.0001)	*F* = 5 (*p* = 0.03)	n.s.
**SWS (%)**	9.1 (6.4)	16.8 (9.8)	3.9 (4.1)	8.7 (7.3)	*F* = 15.2 (*p* < 0.0001)	*F* = 94 (*p* < 0.0001)	*F* = 5 (*p* = 0.03)
**REM (%)**	23.4 (4.9)	18.7 (6.7)	21.8 (4.4)	17 (6.4)	n.s.	*F* = 45.6 (*p* < 0.0001)	n.s.
**NREM (min)**	301.9 (35.6)	276.5 (51.5)	297.2 (39.9)	252.0 (51.9)	n.s.	*F* = 28.3 (*p* < 0.0001)	n.s.

*Note. Untransformed mean (standard deviation)*.

### All-Night Spindle Characteristics

**Figure 1:**
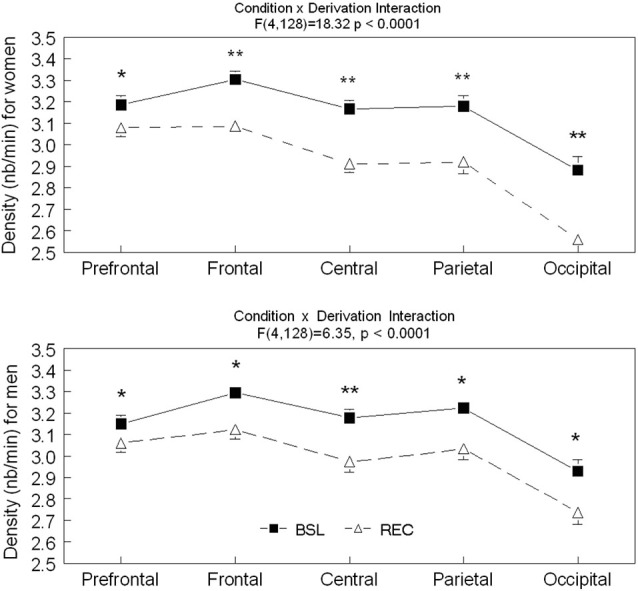
**Spindle density is shown in all derivations for BSL (black squares) and REC (open triangle; mean ± standard error of mean) for women and men**. Simple effects analyses showed significant interactions (*p* < 0.0001) between sleep condition and all derivations for both sexes. (Women—Prefrontal: *F*_(1,59)_ = 36.64; Frontal: *F*_(1,59)_ = 139.71; Central: *F*_(1,59)_ = 165.14; Parietal: *F*_(1,59)_ = 89.87 and Occipital: *F*_(1,59)_ = 121.42; Men—Prefrontal: *F*_(1,59)_ = 16.56; Frontal: *F*_(1,59)_ = 54.48; Central: *F*_(1,59)_ = 72.58; Parietal: *F*_(1,59)_ = 36.41 and Occipital: *F*_(1,59)_ = 46.57). Stars indicate significant differences between BSL and REC in both age groups (for women and men, ES: **>0.7; *<0.7).

Significant interactions between sleep conditions and derivations were found for spindle density in men (*F*_(4,128)_ = 6.35, *p* < 0.0001) and in women (*F*_(4,128)_ = 18.32, *p* < 0.0001; see Figure 1). For both men and women, spindle density was lower in REC sleep compared to BSL sleep in all derivations. The effect was stronger in the central area and weaker in the prefrontal region. No significant effect of age or interaction between age groups and sleep conditions was found for spindle density in men (interaction: *F*_(1,25)_ = 0.24, *p* = 0.63; age only: *F*_(1,25)_ = 0.79, *p* = 0.38) or in women (interaction: *F*_(1,32)_ = 1.21, *p* = 0.28; age only: *F*_(1,32)_ = 1.59, *p* = 0.22).

**Figure 2:**
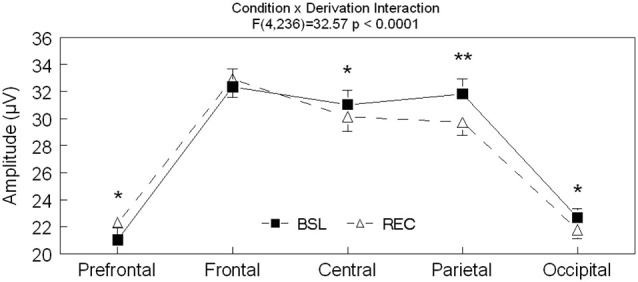
**Spindle amplitude is shown in all derivations for BSL (black squares) and REC (open triangle; mean ± standard error of mean)**. Simple effect analyses showed significant interactions between sleep condition and derivations (Fpz: *F*_(1,59)_ = 18.3, *p* < 0.0001; Fz: *F*_(1,59)_ = 2.4, *p* = 0.12; Cz: *F*_(1,59)_ = 14.4, *p* < 0.0001; Pz: *F*_(1,59)_ = 47.6, *p* < 0.0001 and Oz: *F*_(1,59)_ = 11.8, *p* = 0.001). Stars indicate significant differences between BSL and REC in both age groups (ES: **: 0.447, *: [0.166–0.237]).

**Figure 3:**
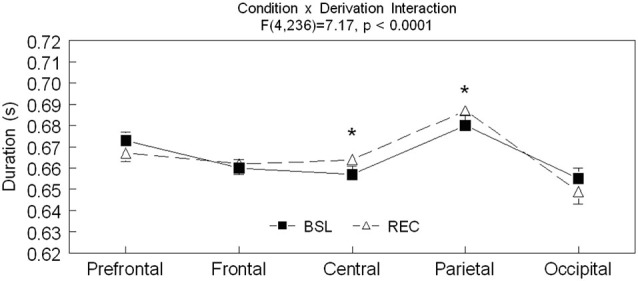
**Spindle duration is shown in all derivations for BSL (black squares) and REC (open triangle; mean ± standard error of mean)**. Simple effect analyses showed significant interactions between sleep condition and derivations (Prefrontal: *F*_(1,59)_ = 3.9, *p* = 0.54; Frontal: *F*_(1,59)_ = 0.41, *p* = 0.53; Central: *F*_(1,59)_ = 6.65, *p* = 0.01; Parietal: *F*_(1,59)_ = 4.46, *p* = 0.04; and Occipital: *F*_(1,59)_ = 2.66, *p* = 0.11). Stars indicate significant differences between BSL and REC in both age groups (ES: *: [0.7–0.10]).

**Figure 4:**
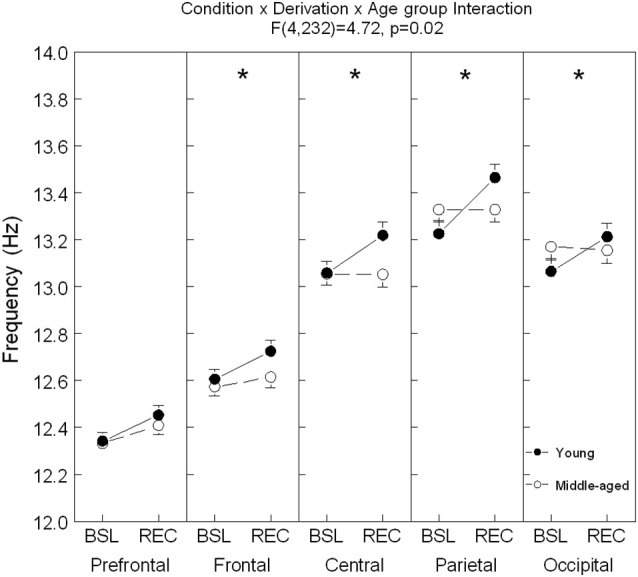
**Spindle frequency is shown in all derivations for young (black dots) and middle-aged (open dots/circle) subjects (mean ± standard error of mean)**. Simple effects analyses showed significant interactions between age condition and sleep condition for each derivation except in the prefrontal area (Frontal: *F*_(1,59)_ = 4.0, *p* = 0.05; Central: *F*_(1,59)_ = 9.73, *p* = 0.003; Parietal: *F*_(1,59)_ = 12.44, *p* = 0.001; and Occipital: *F*_(1,59)_ = 5.74, *p* = 0.02). For young subjects: ES = [0.13–0.32]; for all derivations and for older subjects: ES = 0.20 for Fp1. Stars indicate significant differences between BSL and REC for young subjects (*: *p* < 0.0001).

Significant interactions between sleep conditions and derivations were also found for spindle amplitude (*F*_(4,236)_ = 32.57, *p* < 0.0001) and spindle duration (*F*_(4,236)_ = 7.17, *p* < 0.0001; see Figures 2–4 for *post hoc* analyses). Compared to BSL sleep, spindle amplitude was higher during REC sleep for the prefrontal area but lower for central, parietal, and occipital areas. Finally, compared to REC sleep, spindles lasted longer only in the central and parietal areas in BSL sleep. No significant effect of age groups (*F*_(1,59)_ = 2.0, *p* = 0.16) or interaction between age groups and sleep conditions (*F*_(1,59)_ = 0.06 *p* = 0.81) were found for spindle amplitude. For spindle duration, a main effect of age groups was found (*F*_(1,59)_ = 11.5, *p* = 0.001) with no significant interaction between age groups and sleep conditions (*F*_(1,59)_ = 0.49, *p* = 0.49). Hence, middle-aged subjects showed shorter spindle duration compared to young subjects.

A significant interaction between age groups, sleep conditions and derivations was found for spindle frequency (*F*_(4,236)_ = 4.72, *p* = 0.02; see Figure 4 for contrast analyses). In comparison to BSL sleep, an increase of spindle frequency was observed during REC sleep for young subjects in all derivations, whereas in the middle-aged subjects, spindle frequency was higher only in the prefrontal area.

### Spindles Characteristics and Sleep Efficiency

No significant correlations were found between spindle density, frequency and amplitude at BSL and change in sleep efficiency from BSL to REC sleep (absolute change and percent of change) for young subjects. Only a few moderate counterintuitive negative correlations were found between spindle density in the prefrontal area and the decrease of sleep efficiency in the middle-aged subjects (absolute change and % of change: *r* = −0.46, *p* < 0.01) and in both age groups combined (absolute change: *r* = −0.37, *p* < 0.01; % of change: *r* = −0.36, *p* < 0.01).

## Discussion

Young and middle-aged adults showed comparable differences in spindle density, spindle amplitude and spindle duration during REC sleep compared to BSL sleep. Only spindle frequency showed a differential effect of age between BSL and REC sleep. Although our results illustrated a marked reduction of sleep efficiency during the day associated with aging, spindle characteristics were not linked with the ability to maintain REC sleep.

In our study, during REC sleep compared to BSL sleep, homeostatic sleep propensity was higher (due to sleep loss) and circadian wake propensity increased (due to daytime sleep). Studies evaluating the circadian modulation of sleep spindles have reported higher spindle frequency during daytime sleep as compared to nighttime (Wei et al., 1999; Knoblauch et al., 2003b, 2005). On the other hand, studies showed a reduction in spindle frequency under higher compared to lower sleep homeostatic pressure in young participants (Knoblauch et al., 2003a). During REC sleep, young subjects showed faster spindle frequency compared to BSL sleep over all derivations. This result suggests that in young subjects, the enhancement of spindles frequency by the circadian modulation during daytime overrides the homeostatic pressure for a reduction in spindle frequency induced by the 25-h sleep deprivation. In the middle-aged participants, faster spindle frequency during REC sleep was observed only in the prefrontal area. This observation supports a previous study that showed an age-related reduction in time-of-day modulation of spindle frequency using a 40-h multiple-nap paradigm under constant-routine conditions (Knoblauch et al., 2005).

In the present study, spindle density was lower in REC sleep compared to BSL in all derivations, but this decrease was more prominent in central and frontal areas. These results confirm a previous study, which showed lower spindle density, especially in the frontal derivation, after a 40-h sleep deprivation in young subjects (Knoblauch et al., 2003a). Our results are also congruent with studies showing that spindle incidence and density are lower during daytime compared to night-time sleep (Wei et al., 1999; Knoblauch et al., 2005). Compared to BSL sleep, women showed a stronger decrease in spindle density in the central derivation than men. Higher sigma power and spindle density in women compared to men has been reported in previous studies (Gaillard and Blois, 1981; Carrier et al., 2001; Huupponen et al., 2002; Lafortune et al., 2014). However, no studies have yet evaluated whether homeostatic and circadian modulations of sleep spindles differ between men and women.

Compared to BSL sleep, spindle amplitude was higher during REC sleep for the prefrontal area but lower for central, parietal and occipital areas. These results do not support one previous study, which reported higher spindle amplitude in central, parietal and occipital areas during nocturnal sleep after a 40-h sleep deprivation (Knoblauch et al., 2003a). However, circadian studies showed lower spindle amplitude when sleep is initiated at a circadian time corresponding to daytime (Wei et al., 1999; Knoblauch et al., 2005). Hence, circadian modulation of spindle amplitude probably explains the decrease in spindle amplitude in central, parietal and occipital derivations during daytime REC sleep in our study.

Finally, compared to REC sleep, spindles lasted longer only in the central and parietal areas in BSL sleep. No change in spindle duration was previously reported in nocturnal recovery sleep after a 40-h sleep deprivation (Knoblauch et al., 2003a). Studies evaluating the circadian modulation of spindle duration found conflicting results. One forced desynchrony study reported shorter spindle duration in the central derivation when sleep was initiated at a circadian time corresponding to daytime compared to night-time (Wei et al., 1999), whereas a 40-h nap study showed longer spindle duration in the frontal derivation but shorter duration in the parietal derivation when naps occurred during daytime compared to night-time (Knoblauch et al., 2005).

Middle-aged subjects showed lower sleep efficiency when compared to younger subjects. No significant positive relationship was found between sleep spindles characteristics during the BSL night and change in sleep efficiency between BSL and REC. Our study suggests that individual spindle characteristics do not predict the ability to override the circadian waking signal after sleep loss. Similarly, Knoblauch et al. (2005) did not observe any relationship between the day-night difference in spindle frequency and the day-night difference in wake time. Our results are also in line with our previous results showing no relationship between SW and change in sleep efficiency between BSL and REC sleep (Lafortune et al., 2012). Taken together, these results indicate that individual characteristics in NREM sleep oscillations do not predict the increased wakefulness during daytime recovery sleep. Further studies should aim at understanding the mechanisms that explain the greater sensitivity in older individuals to circadian challenges.

## Conflict of Interest Statement

The authors declare that the research was conducted in the absence of any commercial or financial relationships that could be construed as a potential conflict of interest.
